# Supplementary material for: Polarized α-synuclein trafficking and transcytosis across brain endothelial cells via Rab7-decorated carriers
Source: Fluids Barriers CNS. 2022 May 30;19:37. doi: 10.1186/s12987-022-00334-y (PMC9150364; doi:10.1186/s12987-022-00334-y)
Supplement: Supplementary file 1 — Additional file 1: Table S1. Table of primary antibodies. Table S2. Table of secondary antibodies. Figure S1. Transcytosis of monomeric antibodies. Figure S2. Effect on tight junctions. Figure S3. α-syn trafficking. Figure S4. α-syn not endocytosed. [file 12987_2022_334_MOESM1_ESM.pdf]

Additional file 1

Polarized α-synuclein trafficking and transcytosis across Brain Endothelial Cells via Rab7-decorated carriers

Parvez Alam[1,2,4,†], Mikkel R. Holst[1, †], Line Lauritsen[3], Janni Nielsen[2], Simone S. E. Nielsen[1], Poul Henning Jensen[1], Jonathan R. Brewer[3], Daniel E. Otzen[2*], Morten S. Nielsen[1*]

[1]Department of Biomedicine, Faculty of Health, Aarhus University, Aarhus C, Denmark

[2]Interdisciplinary Nanoscience Center (iNANO), Aarhus University, Aarhus C, Denmark

[3]Department of Biology, University of Southern Denmark, Campusvej 55, 5230 Odense, Denmark

[4]Current affiliation: LPVD, Rocky Mountain Laboratories, NIAID, NIH, Hamilton, MT, 59840, USA

[†]Equal contributors

Address and Email for correspondence: Morten S. Nielsen, Department of Biomedicine, Faculty of Health, Aarhus University, Aarhus C, Denmark (mn@biomed.au.dk) or Daniel E. Otzen, iNANO, Aarhus University, Aarhus C, Denmark (dao@inano.au.dk)

Supplementary Table 1: List of primary antibodies

Target protein	Antibody	Manufacturer	Cat. No.
α-synuclein	Rabbit polyclonal anti α-syn (ASY-1)	Poul Henning Jensen laboratory	
α-synuclein	Mouse monoclonal anti α-syn		610787
EEA1	Mouse polyclonal anti EEA1	BD biosciences	610547
Caveolin 1	Rabbit polyclonal anti cav1	st. John's lab	STJ92051
Rab 7	Mouse monoclonal anti Rab7	Abcam	ab50533
VPS35	Goat polyclonal anti VPS35	Everest Biotech	EB06268
Clathrin	Mouse anti clathrin	Lundbeck, Denmark	
Rab 8	Rabbit monoclonal anti Rab8	Cell signaling	CST-6975T
Claudin 5	Mouse monoclonal anti claudin 5	Thermo Fisher	35-2500
ZO1	Rabbit polyclonal anti ZO1	Invitrogen	61-7300

Supplementary Table 2: List of secondary antibodies

Applied for	Antibody	Manufacturer	Cat. No.
Immunofluorescence labelling of α-syn	Donkey-Anti mouse Alexa 488	Invitrogen	A21202
Immunofluorescence labelling of VPS35	Donkey-anti Goat Alexa 647	Molecular probes	A21082
Immunofluorescence labelling of Rab8a, caveolin 1	Goat-anti rabbit Alexa 647	Invitrogen (Molecular probes)	A21244
Immunofluorescence labelling of α-syn	Donkey-Anti rabbit Alexa 488	Invitrogen (Molecular probes)	A21206
Immunofluorescence labelling of EEA, Rab7, clathrin	Goat-anti mouse Alexa 647	Invitrogen (Molecular probes)	A21235
Immunofluorescence labelling of α-syn	Donkey Anti Rabbit STRAR ORANGE	Invitrogen	A16031/1 mg Abberior Star Orange-NHS ester
Immunofluorescence labelling of Rab7	Goat Anti Mouse STAR RED	Abberior, GmbH	STRED-1001-500UG
Immunofluorescence labelling of VPS35	D Anti G STAR RED	Abberior. GmbH	TRED-1055-500UG

Supplementary figures

Fig. S1

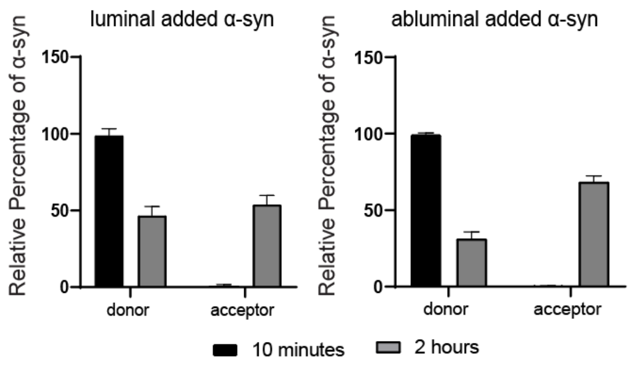

Supplementary figure 1: Relative distribution of donor and acceptor monomeric α-syn depending on transport direction and time. In relation to main figure 2, bar plots show mean values of three independent ELISA measurements, error bars are standard deviations.

Fig. S2

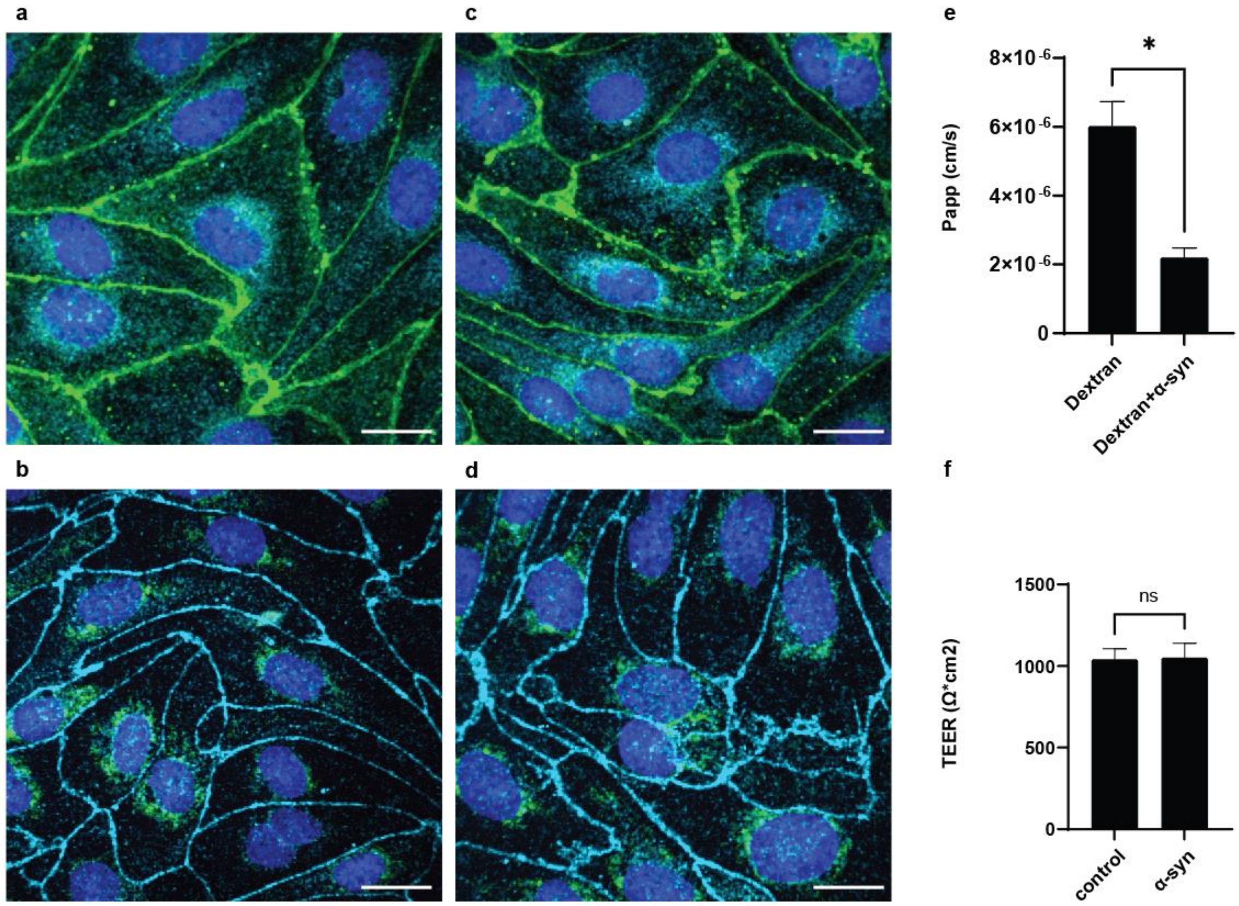

Supplementary figure 2: Effect of α-syn treatment on tight junction protein localization and barrier tightness. Representative micrographs of immunostainings for tight junction marker proteins after 10 minutes (a and b) and two hours (c and d) luminal treatment with 100 nM α-syn. Representative micrographs (a) and (b) Claudin5 (green) and α-syn (turquoise). Representative micrographs (b) and (d) α-syn (green) and ZO-1 (turquoise). Scale bars show 15 µm. Bar plot € show mean values from three independent measurements of apparent permeability coefficient (Papp cm/s) of 14 kD FITC-dextran with or without 100 nM α-syn, error bars are standard deviations. Bar plot (f) show mean values from three independent measurements of trans-endothelial cell resistance measured after two hours with or without 100 nM α-syn, error bars are standard deviations. The statistical tests used were paired t tests (ns = no significance and *P < 0.05). According to the measurements in (e) monomeric α-syn inhibited the permeability of 14 kD FITC-dextran whereas the trans-endothelial cell resistance was unaffected by monomeric α-syn (f).

Fig. S3

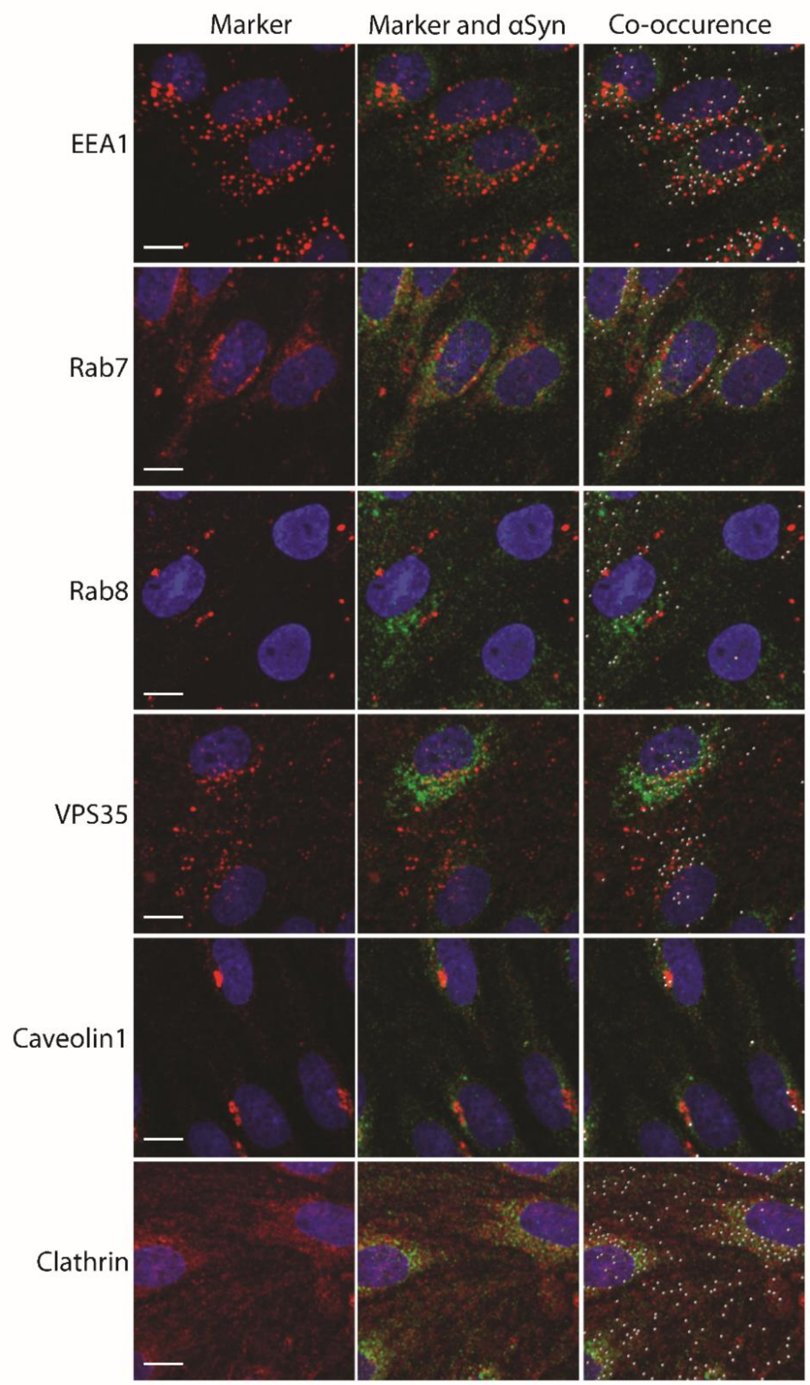

Supplementary figure 3: Representative stains for α-syn co-occurrence with trafficking markers. Representative Maximum projected 3D stacks from confocal micrographs of α-syn monomer treated pBECs on filters with α-syn added for two hours to the luminal side. Green show α-syn monomer stain, blue is Hoechst stain and red is the indicated marker stain. Right micrograph in panels shows segmented IMARIS spots of colocalization analysis between α-syn and marker channels with white points indicating co-occurence. Scale bars show 10 μm.

Fig. S4

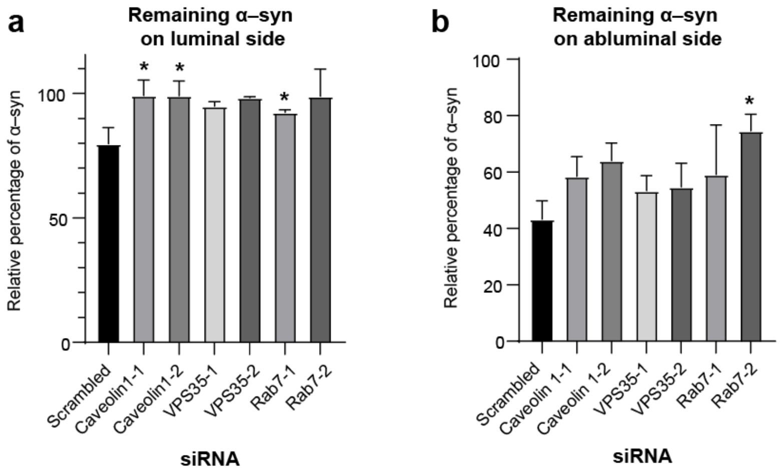

Supplementary figure 4: Effect of intracellular trafficking machinery on α-syn transport through the BBB model. In relation to figure 5, relative percentage of remaining α-syn (after two hour chase) in donor chamber from cells pretreated with indicated siRNA. Remaining α-syn added to luminal (a) and abluminal (b) side of the BBB model, respectively. Bar plots in (a) and (b) show mean values of three independent ELISA measurements, error bars show standard deviations. Statistical difference was tested using an ordinary one-way ANOVA followed by Dunnett's multiple comparisons test (*$P < 0.05$ compared to scrambled control).
